# The Activation of Phosphatidylserine/CD36/TGF-*β*1 Pathway prior to Surgical Brain Injury Attenuates Neuroinflammation in Rats

**DOI:** 10.1155/2020/4921562

**Published:** 2020-08-11

**Authors:** Lei Huang, Hailiang Tang, Prativa Sherchan, Cameron Lenahan, Warren Boling, Jiping Tang, John H. Zhang

**Affiliations:** ^1^Department of Neurosurgery, Loma Linda University, 11234 Anderson Street, Loma Linda, CA 92354, USA; ^2^Department of Physiology and Pharmacology, Loma Linda University, 11041 Campus Street, Loma Linda, CA 92354, USA; ^3^Department of Neurosurgery, Huashan Hospital, Fudan University, 12 Wulumuqi Middle Rd, Jing'an District, Shanghai 201206, China; ^4^Burrell College of Osteopathic Medicine, Las Cruces, NM 88003, USA; ^5^Department of Anesthesiology, Loma Linda University, 11234 Anderson Street, Loma Linda, CA 92354, USA

## Abstract

Neuroinflammation plays an important pathological role in experimental surgical brain injury (SBI). Apoptotic associated with phosphatidylserine (PS) externalization promotes anti-inflammatory mediator TGF-*β*1 release. In the present study, we investigated the anti-neuroinflammation effect of PS liposome or isoflurane pretreatment via PS/CD36/TGF-*β*1 signaling in a rat model of SBI. A total of 120 male Sprague-Dawley rats (weighing 280-330 gms) were used. SBI was induced by partial right frontal lobe corticotomy. Intranasal PS liposome or isoflurane inhalation was administered prior to SBI induction. CD36 small interfering RNA (siRNA) was administered intracerebroventricularly. Recombinant Annexin V protein (rAnnexin V) was delivered intranasally. Post-SBI assessments included neurological tests, brain water content, Western blot, and immunohistochemistry. Endogenous CD36 protein levels but not TGF-*β*1 was significantly increased within peri-resection brain tissues over 72 h after SBI. SBI rats were associated with increased brain water content surrounding corticotomy and neurological deficits. PS liposome pretreatment significantly reduced brain water content and improved some neurological deficits at 24 hours and 72 hours after SBI. PS liposome increased CD36 and TGF-*β*1 protein levels, but decreased IL-1*β* and TNF*α* protein levels in peri-resection brain tissues at 24 hours after SBI. CD36 siRNA or rAnnexin V partially countered the protective effect of PS liposome. Isoflurane pretreatment produced similar antineuroinflammation and neurological benefits in SBI rats partially by upregulating CD36/Lyn/TGF-*β*1 signaling. Collectively, our findings suggest that the activation of PS/CD36/TGF-*β*1 pathway by PS liposome or isoflurane prior to SBI could attenuate neuroinflammation and improve neurological outcomes in rats. PS liposome or isoflurane pretreatment may serve as an effective preventive strategy to minimize the brain injury caused by neurosurgical procedures in patients.

## 1. Introduction

Neurosurgical procedure itself has been shown to cause surgical brain injury (SBI) that contributes to the postoperative complications of brain edema, ischemia, and intracranial hematoma as well as neurological deficits [1, 2]. There are 13-27% incidences of major postsurgical complications in patients undergoing intracranial surgeries [3]. Neuroinflammation plays an important role in the pathology of SBI. In experimental setting of SBI, neuroinflammation was characterized as microglial activation, peripheral immune cell infiltration, and increased proinflammatory cytokines within peri-resection brain tissues, which contributed to brain edema, brain tissue oxidative stress, and poor neurobehavioral performance [4–7]. Therapeutic strategies targeting neuroinflammation would minimize postoperative neurological deficits in patients undergoing neurosurgical procedures.

Apoptotic cell-triggered phagocytic signaling is anti-inflammatory. It is initiated by phosphatidylserine (PS) externalization to the outside layers of apoptotic cell membranes and PS recognition by phagocytic receptors of immune cells [8, 9]. Apoptotic-associated PS receptor activation suppressed the elevation of proinflammatory mediators but induced anti-inflammatory mediators release including TGF-*β*1 [9–11]. In vitro study, apoptotic cells induced TGF-*β*1 synthesis in macrophage by activating CD36/Lyn, ERK1/2 pathway in vitro [12]. Administration of PS liposome 24 hours prior to and at the beginning of ischemia increased anti-inflammatory TGF-*β*1 level, suppressed the induction of proinflammatory markers, and promoted retinal neuron survival after ischemic injury [13]. Interestingly, isoflurane also has been shown to protect renal proximal tubules by PS initiated-TGF-*β*1 release in vitro [14, 15]. TGF-*β*1 suppresses inflammation and promotes survival in adult central nervous system [16]. Thus, activation apoptotic mimicry phagocyte signaling may serve as a promising preventive neuroprotection strategy by creating an anti-inflammatory microenvironment against subsequent brain injury.

In the present study, we hypothesized that the activation of microglial phosphatidylserine/CD36/TGF-*β*1 signaling pathway prior to brain resection will reduce neuroinflammation and improve neurological outcomes in a rat model of SBI.

## 2. Material and Methods

### 2.1. Animals

All animal procedures in this study are complied with the NIH Guide for the Care and Use of Laboratory Animals. The experimental protocol was approved by the Institutional Animal Care and Use Committee (IACUC) at Loma Linda University. Adult male Sprague-Dawley (SD) rats, weighing 280-330 gms, were used and housed in an animal care facility with a 12-hour light/dark cycle and free access to food and water.

### 2.2. Experimental Design

**Figure 1:**
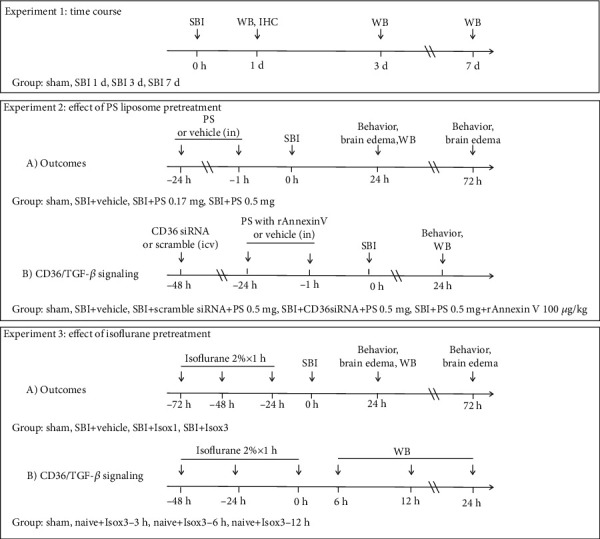
Experimental design and animal groups. SBI: surgical brain injury; WB: Western blot; IHC: immunohistochemistry; PS: phosphatidylserine; rAnnexin V: recombinant Annexin V; in: intranasal; icv: intracerebroventricular injection; Iso: isoflurane.

A total of one hundred and twenty rats were used. The study design was shown in Figure 1.

Experiment 1 assessed the temporal expressions of endogenous CD36 and TGF-*β*1 within peri-resection brain tissues at 1, 3, and 7 days after SBI. A total of sixteen rats were randomly divided into four groups of sham, SBI-1d, SBI-3d, and SBI-7d for Western blot measurement (*n* = 4/group). Immunohistochemistry was used to assess the CD36 localization on microglia at 1 d after SBI (*n* = 2).

Experiment 2 evaluated the neuroprotective effects of PS liposome pretreatment in the rat model of SBI. (A) Testing the effect on brain edema and neurological deficit after SBI. Firstly, we evaluated the dose effects on brain edema and neurological deficits at 24 hours after SBI. A total of 24 rats were randomized into the four groups: sham (*n* = 6), SBI+vehicle (*n* = 6), SBI+PS 0.17 mg/rat (*n* = 6), and SBI+PS 0.5 mg/rat (*n* = 6). PS liposome (10 mg/mL in PBS, Encapsula NanoSciences, Brentwood, TN) or same volume of phosphate-buffered saline (PBS) as vehicle was administrated via intranasal route 1 day and 1 hour prior to the SBI. Based on the results at 24 hours post-SBI, the most effective dose of PS liposome (0.5 mg/rat) was selected for the following experiments. Secondly, a total of 18 rats were randomized into the three groups: sham (*n* = 6), SBI+vehicle (*n* = 6), and SBI+PS 0.5 mg/rat (*n* = 6), in which we evaluated the effect of PS liposome pretreatment on the outcomes of brain edema and neurological deficits at 72 hours post-SBI. Lastly, we evaluated the effects of PS liposome pretreatment on TGF-*β*1 singaling-mediated inflammation. Another set of 18 animals were randomized into the three groups: sham (*n* = 6), SBI+vehicle (*n* = 6), and SBI+PS 0.5 mg/rat (*n* = 6). At 24 hours after SBI, brain tissues were collected for Western blot assessments of TGF-*β*1/IL-1*β*/TNF*α*. These brain samples were also used for the following experiment. (B) Investigating whether the effects of PS liposome pretreatment were mediated through activation of CD36/TGF-*β*1 singaling. Additional 18 rats were randomized to three additional groups of SBI+PS 0.5 mg/rat+CD36 siRNA (*n* = 6), SBI+PS 0.5 mg/rat+scramble siRNA (*n* = 6), and SBI+PS 0.5 mg/rat+rAnnexin V 100 *μ*g/kg (*n* = 6). The CD36 siRNA (Accell SMART pool, Thermo Fisher Scientific, Pittsburgh, PA), 500 pmol dissolved in 2 *μ*L RNase free water, was administered via intracerebroventricular (i.c.v) route 48 hours before SBI. The 2 *μ*L scramble siRNA (siGENOME nontargeting siRNA, Thermo Fisher Scientific, Pittsburgh, PA) was administered in control groups. Recombinant Annexin V (rAnnexin V, eBioscience, Inc., San Diego, CA), a protein with high affinity to PS, was administered with PS liposome via intranasal route to minimize the binding between PS and CD36 receptor.

Experiment 3 assessed the neuroprotective effects of isoflurane pretreatment via CD36/TGF-*β*1 signaling activation. (A) Evaluating the effect on brain edema, neurological deficit, and neuroinflammation. Twelve rats in three additional groups were added: SBI+isoflurane ×1 d (*n* = 6), SBI+isoflurane ×3 d (*n* = 6). Isoflurane (2% in 3 L/min 100% oxygen and 7 L/min medical air mixture) for 1 hour was inhaled by the rats that were placed in a plastic treatment chamber. For isoflurane ×1 d group and isoflurane ×3 d group, isoflurane pretreatment was administered to rats at 1 day or 3 days/2 days/1 day prior to SBI induction, respectively. (B) Investigating the effects of isoflurane pretreatment on CD36/Lyn/TGF-*β*1 singaling. Twelve naïve rats were randomized into the following three groups with *n* = 4/group: naïve+isoflurane×3 d-6 h, naïve +isoflurane×3 d-12 h, and naïve+isoflurane×3 d-24 h.

### 2.3. Surgical Brain Injury Rat Model

The SBI was induced in rats as previously described [5, 17]. Briefly, rats were anesthetized with ketamine/xylazine (80/20 mg/kg). After rats were placed in a prone position on a stereotactic frame, a midline skin was incised, and skull was exposed. Using a microdrill, a 5 × 5 mm square craniotomy was performed on the right side of frontal bone with intact dura, whose bottom margin was 1 mm to coronal and the left margin was 2 mm lateral to the sagittal suture. In SBI animals, the dura was incised, and the right frontal lobe was partially removed along the margins of craniotomy with the depth of resection reaching the base of the skull. Upon completing the brain tissue resection, hemostasis was achieved using repeated normal saline irrigation followed by intraoperative packing. After the hemostasis was ensued, the incision was sutured. Sham surgeries underwent the same craniotomy in the absence of the subsequent dura incision and partial right frontal lobectomy. Body temperature was controlled using thermal heat lamps throughout surgery. After surgery, the animals were kept in the recovery cage and monitored until recovering from anesthesia.

### 2.4. Intranasal Administration

Intranasal administration of PS liposome in PBS (0.17 mg or 0.5 mg) and PBS or rAnnexin V (100 *μ*g/kg) was performed as previously described [18]. Briefly, the rat was anesthetized under 2% isoflurane and placed in supine position on a heating pad. Using a manual pipette, a drop of 8 *μ*L drugs or PBS was administered into each nare with left and right alternation every 2 minutes. The daily intranasal PS liposome pretreatment was completed within 20 minutes.

### 2.5. Intracerebroventricular Injection

For siRNA administration at 24 hours prior to SBI, the i.c.v. injection was performed as previously described [5, 7]. After anesthetized with ketamine/xylazine (80/20 mg/kg), the rats were fixed on a stereotaxic frame in a prone position. A burr hole was made by microdrill on the right parietal skull at coordinates of 1.5 mm posterior and 1.0 mm lateral to the bregma. Through the burr hole, the needle of a 10 *μ*L Hamilton syringe was advanced into the right lateral ventricle at depth of 3.2 mm below the horizontal plane of the bregma. According to the manufacturer's instructions, a total 500 pmol Accell SMART pool rat siRNA CD36 (Dharmacon/Thermo Fisher Scientific, Lafayette, CO, USA) in a 2 *μ*L sterile saline was administered at 0.5 *μ*L/minute into the right ventricle. Four different sequences targeting CD36 were pooled: GGAUGAGCCUACAUUAUGC, GAAUUGGCUUUAAAUAUGU, CUCUUUAUUUUAAGU-AUGU, and GCAUAUUUCGAAAAGAUUA. For negative control, a 2 *μ*L scramble siRNA (Accell nontargeting pool, Dharmacon/Thermo Fisher Scientific, Lafayette, CO, USA) was injected. After completing injection of siRNA CD36/scramble siRNA, the needle was kept *in situ* for an additional 10 minutes and withdrawn slowly to prevent possible backflow. Bone wax was used to seal the burr hole, and the incision was sutured. The rats were kept in the recovery cage and monitor until recovering from anesthesia.

### 2.6. Brain Water Content

Brain edema was assessed using wet weight/dry weight method as previously described [5, 17]. Under deep anesthesia, the rat was quickly decapitated, and the whole brain was dissected into six regions, namely, right frontal, left frontal, right parietal, left parietal, cerebellum, and brain stem. The wet weight of each region was measured immediately, after which the brain tissues were dried in an oven at 105°C for 48 hours for measuring the dry weight. The formula of [(wet weight − dry weight)/wet weight] × 100% was used to calculate the percentage of water content for each brain region [5, 17].

### 2.7. Neurobehavioral Tests

As previously described, the modified Garcia test and beam balance test were performed to accesses post-SBI neurological functions [5, 7, 17] by an investigator (PS) blinded to the information of experimental groups. Briefly, the modified Garcia test assesses the sensorimotor function, consisting of seven individual tests for spontaneous activity, side stroking, vibrissae touch, limb symmetry, climbing, lateral turning, and forelimb walking. The maximum score was 21 with a score range of 0-3 in each test. The beam balance test was to evaluate the total walk distance and time after placing a rat on a 90 cm × 2.25 cm beam for 1 minute. The scores of beam test ranged from 0 to 5. For both tests, the higher scores suggested a better neurological function.

### 2.8. Western Blot Assay

Western blotting was performed as described previously [5, 7]. Briefly, rats were transcardially perfused with cold PBS (pH 7.4). The brains were quickly removed from the skull and dissected into 6 regions of right frontal, left frontal, right parietal, left parietal, cerebellum, and brain stem. Right frontal brain tissues were homogenized in RIPA lysis buffer (Santa Cruz Biotechnology, Inc., Dallas, TX, USA). After 30 minutes of centrifugation at 14,000 g at 4°C, the supernatant was collected as whole-cell protein extract, and the protein concentration was determined using a detergent compatible assay (Bio-Rad, Hercules, CA, USA). The same amounts of protein were loaded and separated by electrophoresis on a sodium dodecyl sulfate polyacrylamide (SDS-PAGE) gel. After the nitrocellulose membrane transfer, the membrane was incubated with the respective primary antibody overnight at 4°C including rabbit polyclonal anti-CD36 (1 : 800, Santa Cruz Biotechnology, Inc, Dallas, TX), rabbit polyclonal anti-TGF*-β*1 (1 : 1000, Abcam, Cambridge, MA), rabbit monoclonal anti-phospho Lyn (p-Lyn, 1 : 1000, Abcam, Cambridge, MA), rabbit monoclonal anti-Lyn (1 : 1000, Cell Signaling Technology, Danvers, MA), rabbit polyclonal anti-IL1*β* (1 : 1000, Abcam, Cambridge, MA), and rabbit polyclonal anti-TNF*α* (1 : 600, Abcam, Cambridge, MA). The same membranes were incubated with primary antibody of goat anti-*β*-actin (1 : 4000 Santa Cruz Biotechnology, Dallas, Texas) for a loading control. Subsequently, membranes were incubated with corresponding secondary antibodies (1 : 4000, Santa Cruz Biotechnology, Inc, Dallas, TX) for 1 hour at room temperature. Western blot bands were further exposed to an X-ray film using a chemiluminescence reagent kit (ECL Plus, Amersham Biosciences, Arlington Heights, IL) and quantified by Image J software (NIH, USA).

### 2.9. Immunohistochemistry

Immunohistochemistry was performed at 24 hours after SBI as described previously [5, 7]. Briefly, the deep anesthetized rats were intracardially perfused with ice-cold PBS and 10% formalin. The whole brains were removed from skull and postfixed in 10% formalin at 4°C for 24 hours followed by the dehydration in 30% sucrose in PBS. The brains were sectioned into 10 *μ*m thick coronal slices using a cryostat (CM3050S; Leica Microsystems, Bannockburn, IL). The slices were coincubated with the primary antibodies of rabbit polyclonal anti-CD36 (1 : 100, Santa Cruz Biotechnology, Inc, Dallas, TX) with goat polyclonal anti-ionized calcium-binding adapter molecule 1 (IBA1, 1 : 200, Abcam, Cambridge, MA) overnight at 4°C, followed by corresponding FITC-conjugated and Texas Red-conjugated secondary antibodies (1 : 400, Jackson ImmunoResearch, West Grove, PA) for 2 hours at room temperature. The fluorescence staining was visualized with a fluorescence microscope (Olympus BX51).

### 2.10. Statistics

The data was presented as the Mean ± SEM. For 24 hours, neurological score analysis, data collected from 24 hours and 72 hours brain edema outcomes, and the Western blot studies were all used with *n* = 17 in groups of sham, SBI+vehicle, SBI+PS 0.5 mg/rat, and SBI + Isox3; *n* = 12 in SBI+Iso x1 group. Statistical differences for each time point was analyzed using the one-way ANOVA followed by the Student-Newman-Keuls post hoc test. A *p* value less than 0.05 was considered statistically significant.

## 3. Results

All sham-operated rats survived. The overall mortality of SBI was 4%. The mortality was not significantly different among the experimental groups.

### 3.1. Time Course of Endogenous Levels of CD36 and TGF-*β*1 after SBI

**Figure 2:**
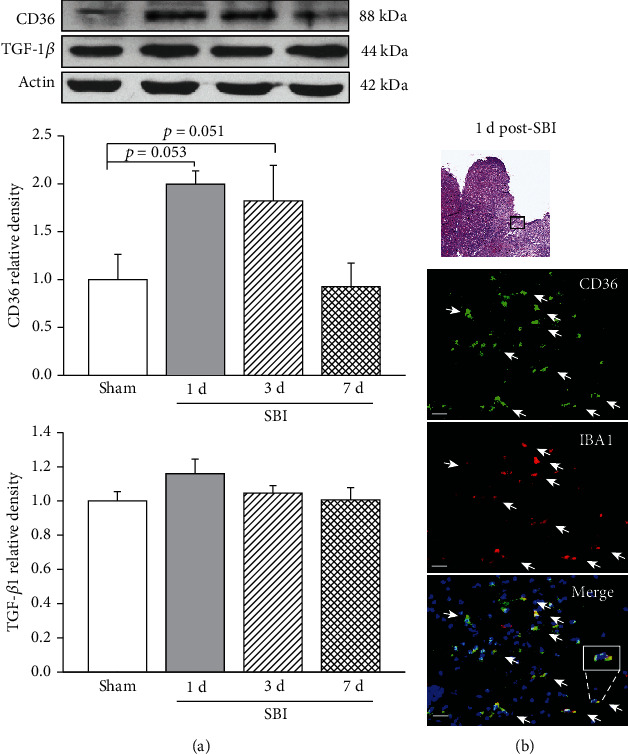
Time course of CD36 and TGF-*β*1 expressions within peri-resection region of brain. (a) Endogenous CD36 level but not TGF-*β*1 level was increased at 1 d and 3 d after SBI. *n* = 4/group, *p* = 0.029; (b) representative microphotograph showed that CD36 receptors were colocalized with IBA1-positive microglia cells. Scale bar = 25 *μ*m.

Western blot assay showed that brain CD36 protein levels were increased within peri-resection region at 1 day (*p* = 0.053) and 3 days (*p* = 0.051) after SBI (Figure 2(a)). There were no significant changes in the protein levels of TGF-*β*1 over 7 days after SBI (*p* > 0.05). Double immunofluorescence staining showed the expressions of CD36 were colocalized with IBA1-positive microglia at 1 day after SBI (Figure 2(b)).

### 3.2. PS Liposome Pretreatment Improved the Outcomes of SBI

**Figure 3:**
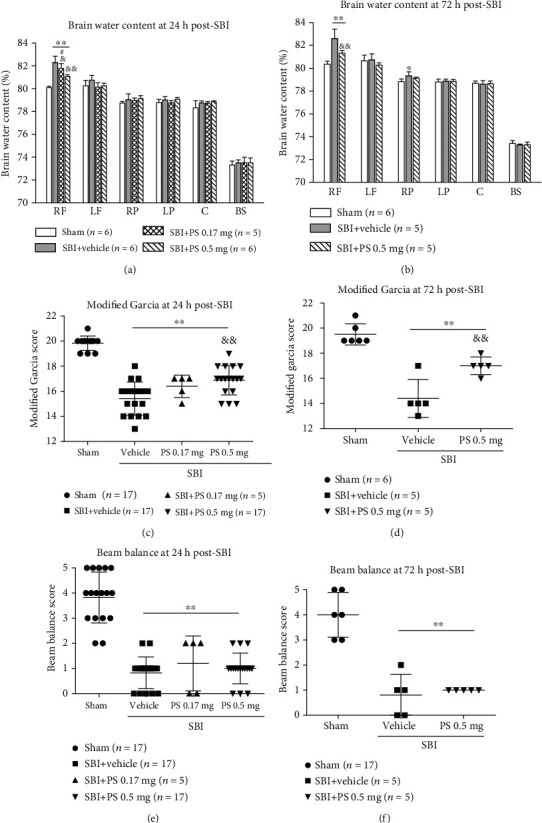
PS liposome pretreatment effects on brain edema and neurological outcomes at 24 h and 72 h post-SBI. PS liposome pretreatment significantly reduced the brain edema at 24 h post-SBI (a). PS 0.5 mg pretreatment improved Garcia scores (c), but not beam balance scores (e). The neuroprotection of PS 0.5 mg pretreatment on brain edema and Garcia scores persisted at 72 h post-SBI (b, d). PS pretreatment did not improve beam balance scores (e, f). ^∗∗^*p* < 0.01 vs. sham; ^&&^*p* < 0.01, ^&^*p* < 0.05 vs. SBI+vehicle, ^#^*p* < 0.05 vs. SBI+PS 0.5 mg. RF: right frontal lobe; LF: left frontal lobe; RP: right parietal lobe; LP: left parietal lobe; C: cerebellum; BS: brain stem.

At 24 and 72 hours after SBI, there were higher brain water content within peri-resection frontal brain tissues and greater neurological deficits than shams (*p* < 0.05, Figures 3(a) and 3(c)). Compared with the vehicle pretreated SBI rats, pretreatment of PS liposome at the dose of 0.17 mg or 0.5 mg significantly reduced brain edema at 24 hours post-SBI although the improved neurological performance of Garcia test was only observed in rats pretreated by PS at the dose of 0.5 mg (Figures 3(a) and 3(c)). The protective effects of PS 0.5 mg was persistent to 72 hours post-SBI including significantly less brain water content and better Garcia neurological scores (*p* < 0.05, Figures 3(b) and 3(d)). Beam balance performance, however, was not improved by PS liposome pretreatment at either of the dosages (*p* > 0.05, Figures 3(e) and 3(f)).

### 3.3. The Effects of PS Liposome Pretreatment on Neuroinflammation at 24 Hours after SBI

**Figure 4:**
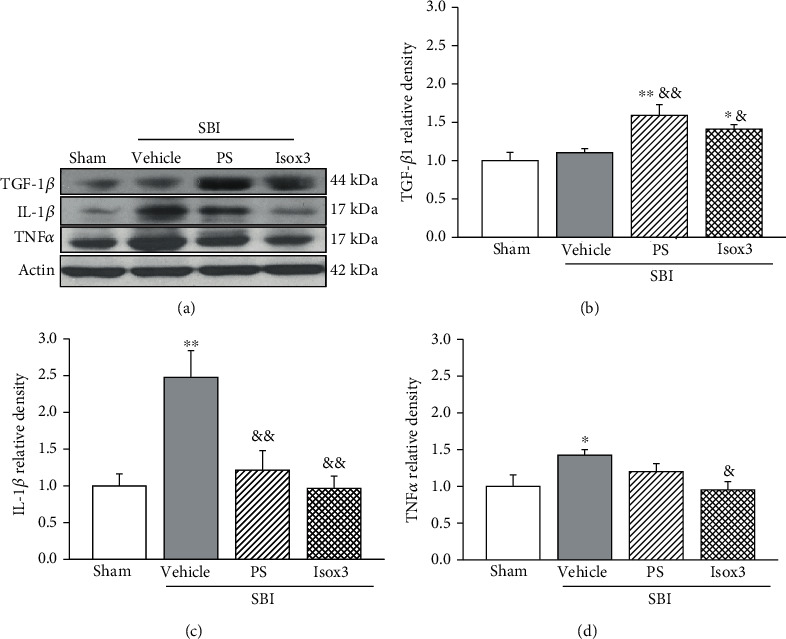
PS liposome or isoflurane pretreatment effects on neuroinflammation within peri-resection region of brain at 24 h post-SBI. Representative Western blot bands (a). Quantitative analysis of Western blot bands showed that PS liposome pretreatment increased TGF-*β*1 level (b) and decreased proinflammatory cytokines level of IL-1*β* and TNF*α* (c, d). Isoflurane pretreatment resulted in significant decreases in IL-1*β* and TNF*α* (c, d) with tendency toward increase in TGF-*β*1 level (b). *n* = 5 − 6/group. ^∗∗^*p* < 0.01, ^∗^*p* < 0.05 vs. sham; ^&&^*p* < 0.01, ^&^*p* < 0.05 vs. SBI+vehicle.

Compared with vehicle-pretreated SBI rats, PS liposome pretreatment significantly reduced the proinflammatory cytokine levels including IL-1*β* and TNF*α* within peri-resection frontal brain tissues at 24 hours post-SBI, which is associated with increased the protein level of TGF-*β*1 (*p* < 0.05, Figure 4).

### 3.4. The Effects of CD36 siRNA or rAnnexin V on the Protective Effects of PS Liposome Pretreatment at 24 Hours after SBI

**Figure 5:**
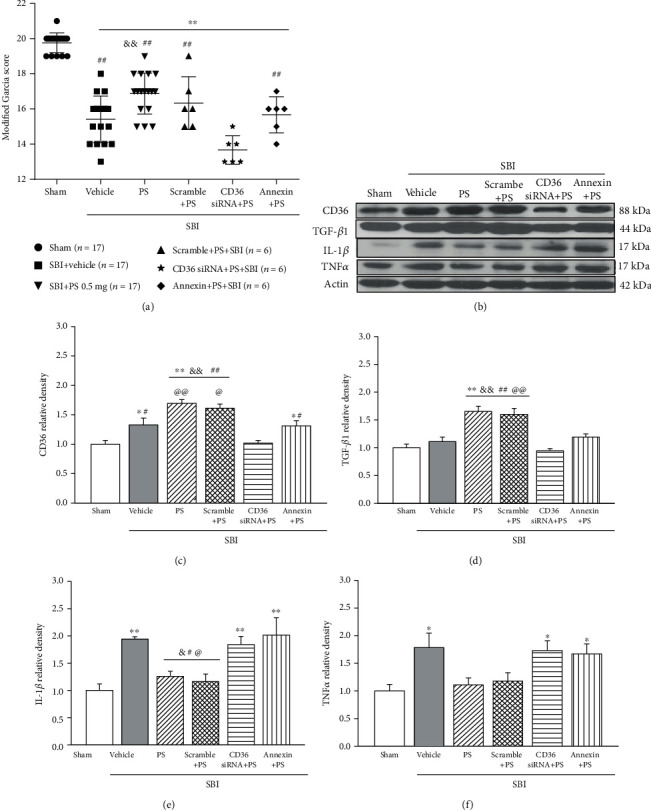
Effects of CD36 siRNA and rAnnexin V on PS liposome pretreatment-induced neuroprotection at 24 h post-SBI. (a) CD36 siRNA or rAnnexin V reversed the Garcia scores improvement associated with the PS liposome pretreatment; (b–f) Western blot assay showed that PS pretreatment significantly increased the level of CD36 and TGF-*β*1, which was associated with less IL-1*β* and TNF*α* expressions. CD36 siRNA or rAnnexin V significantly abolished anti-inflammation associated with PS liposome pretreatment. *n* = 5 − 6/group. ^∗∗^*p* < 0.01, ^∗^*p* < 0.05 vs. sham, ^&&^*p* < 0.01, ^&^*p* < 0.05 vs. SBI+vehicle; ^##^*p* < 0.01, ^#^*p* < 0.05 vs. SBI+CD36 siRNA+PS; ^@@^*p* < 0.01, ^@^*p* < 0.05 vs. SBI+Annexin+PS.

CD36 siRNA but not rAnnexin V significantly reversed the neurobehavioral benefits of PS liposome pretreatment in SBI rats (*p* < 0.05, Figure 5(a)). Western blot assay showed PS pretreatment group was associated with significantly higher protein levels of CD36 and TGF-*β*1, but lower level of proinflammatory cytokine including IL-1*β* and TNF*α* in peri-resection brain tissues, when compared to vehicle group. Such was reversed by CD36 siRNA or rAnnexin V (*p* < 0.05, Figures 5(b)–5(f)).

### 3.5. The Effects of Isoflurane Pretreatment on Brain Edema, Neurological Deficits, and Neuroinflammation after SBI

**Figure 6:**
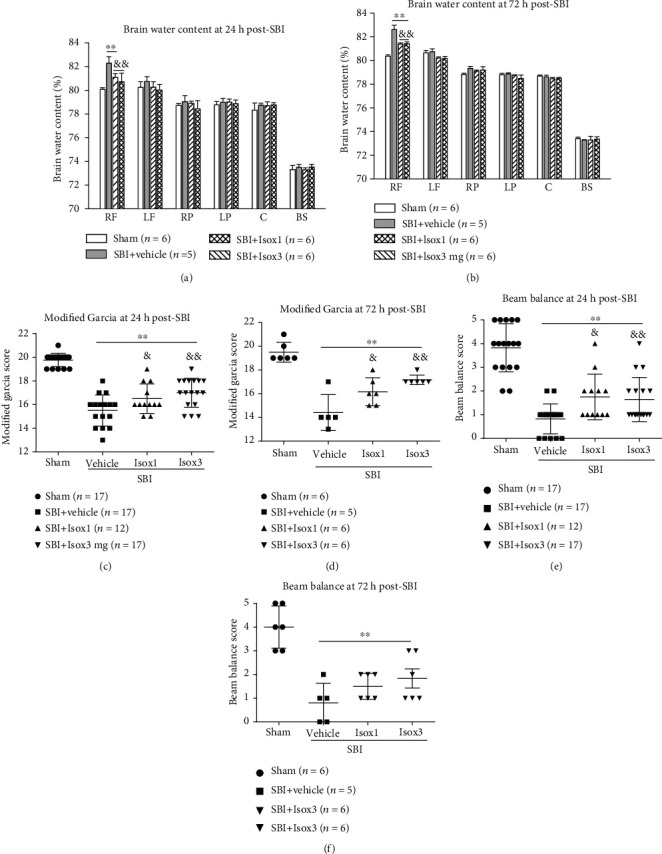
Isoflurane pretreatments regimen of 1 time or 3 times significantly reduced the brain edema and improved neurological outcomes at 24 h post-SBI (a, c, e). The neuroprotection including brain edema attenuation and Garcia neurological score improvement persisted at 72 h post-SBI (b, d, f). ^∗∗^*p* < 0.01 vs. sham, ^&&^*p* < 0.01; ^&^*p* < 0.05 vs. SBI+vehicle.

At 24 hours post-SBI, either one day of isoflurane pretreatment or three days of isoflurane pretreatment significantly reduced the brain edema within the peri-resection brain tissues and improved the performance of Garcia test and beam balance test compared with vehicle-pretreated SBI rats (*p* < 0.05, Figures 6(a), 6(c), and 6(e)). The protective effects of three days of isoflurane pretreatment on brain edema (*p* < 0.05) and Garica test scores (*p* < 0.05) but not beam balance test score (*p* > 0.05) persisted to 72 hours post-SBI (Figures 6(b), 6(d), and 6(f)). Similar to PS liposome pretreatment, isoflurane 3-day pretreatment regimen significantly reduced the proinflammatory cytokine levels including IL-1*β* and TNF*α* (*p* < 0.05) within peri-resection frontal brain tissues at 24 hours post-SBI with a tendency toward increase in TGF-*β*1 level (Figure 4).

### 3.6. The Effects of Isoflurane Pretreatment on CD36/Lyn/TGF-*β*1 Signaling Pathway in Naïve Rats

**Figure 7:**
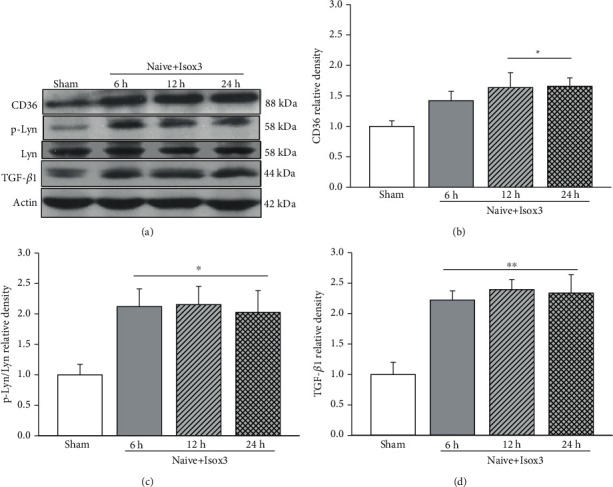
Isoflurane pretreatment upregulated CD36/Lyn/TGF-*β*1 signaling pathway in naïve rats. Representative Western blot bands (a). Western blot results showed that the protein levels of CD36 (b), p-Lyn (c), and TGF-*β*1 (d) significantly increased starting at 6 h, and the elevations maintained at 24 h after pretreatment. *n* = 4/group. ^∗∗^*p* < 0.01, ^∗^*p* < 0.05 vs. sham.

Western blot showed that 1 hour daily for three consecutive days of isoflurane pretreatment significantly increased the protein levels of CD36, p-Lyn, and TGF-*β*1 in frontal lobe brain tissues of naïve rats. The elevation started at 6 hours and persistent to 24 hours after the last episode of isoflurane pretreatment (*p* < 0.05, Figure 7). The upregulation of CD36/Lyn/TGF-*β*1 prior to SBI, at least partially, contributes to the neuroprotection provided by isoflurane pretreatment in SBI rats.

## 4. Discussion

In the present study, we had the following findings: (1) Endogenous CD36 levels were significantly elevated in peri-resection brain tissues at 1 day and 3 days, but not 7 days after partially frontal corticotomy in a rat model of SBI. CD36 expressions were colocalized with microglia. However, the change of endogenous TGF-*β*1 in the peri-resection brain tissues was not significantly change over 7 days post-SBI; (2) PS liposome at a dose of 0.5 mg significantly attenuated SBI-induced brain edema and neurological deficits 24 hours and 72 hours after SBI; (3) PS liposome reduced proinflammatory cytokines release in peri-resection brain tissues 24 hours after SBI. The anti-inflammation effects were associated with increased protein levels of TGF-*β*1; (4) CD36 siRNA or exogenous rAnnexin V partially offset the neuroprotection provided by PS liposome at 24 hours after SBI; (5) Isoflurane pretreatment significantly attenuated SBI-induced brain edema and neurological deficits 24 hours and 72 hours after SBI. Isoflurane pretreatment also reduced proinflammatory cytokines release in peri-resection brain tissues 24 hours after SBI; (6) Isoflurane pretreatment upregulated CD36/Lyn/TGF-*β*1 signaling up to 24 hours prior to SBI. Collectively, these results demonstrated the activation of PS/CD36/TGF-*β*1 signaling pathway by PS liposome or isoflurane pretreatment attenuated neuroinflammation, leading to the improved neurological outcomes in the setting of experimental SBI.

PS is an aminophospholipid located predominantly on the inner leaflet of cell cytoplasmic plasma membrane in normal condition. Due to the loss of membrane phospholipid asymmetry during apoptosis, PS exposes to the outer leaflet of the cytoplasmic plasma membrane and binds by PS recognition receptors of phagocytes including macrophages and microglia [19–21]. The subsequent internalization of apoptotic cells by phagocytes would stimulate phagocytes to produce anti-inflammatory cytokines but inhibit the release of proinflammatory mediators (Fadok et al., 2001; Huynh et al., 2002; Minghetti et al., 2005). CD36 is a member of the class B scavenger receptor family [22] that has been identified as one of PS recognition receptors participating in apoptotic cell recognition and clearance [11, 12, 23, 24]. CD36 activation by apoptotic cells or CD36 antibody has been shown to enhance TGF-*β*1 transcription and the release of TGF-*β*1 protein from macrophages in vitro [12]. In the present study, there was an upregulation of CD36 protein within peri-resection brain tissues at 1 day and 3 days after SBI. Immune cells have shown to be the major source of CD36 in the postischemic brain [25]. After SBI, there were immune cell accumulations including microglia in peri-resection brain tissue [5, 6, 26], which may contribute to CD36 elevation. We found the CD36 receptors colocalize with microglia. To be noted, the previous study showed an elevation of CD36 at 7 days after brain ischemia in mice [27]. In the present study, however, the temporal increase in CD36 expression was not significant at 7 days after SBI. The discrepancy may be due to the different animal model with different pathological process. There was a gradual recovery of neurological function to almost that of presurgery by 7 days after SBI in rats [17], suggesting the potential resolution of immune responses within 1 week in the rat model of SBI we used. Future study is needed to elucidate the exact mechanism. Nevertheless, the endogenous increase in endogenous TGF-*β*1 protein level appeared not to be sufficient after SBI.

Both in vitro and in vivo experiments have demonstrated that PS liposome could mimic the effects of apoptotic cells on phagocytes and exert anti-inflammatory effects [10, 28–30]. The protective role of PS liposome pretreatment has also been shown in a rat model of lipopolysaccharide- (LPS-) induced impairment of long-term potentiation [30]. Intraperitoneal PS liposome pretreatment regimen in a mouse model of retinal ischemia reperfusion injury significantly reduced the proinflammatory genes at 24 hours and improved the retinal neuronal survival at 7 days after reperfusion [13]. Consistently, when we pretreated rats with PS liposome intranasally 24 hours and 1 hour prior to SBI induction, there were significantly decreased brain edema and neurological deficits at 24 hours and 72 hours postinjury. Those post-SBI outcome benefits were associated with increased TGF-*β*1 protein level and decreased proinflammatory cytokines IL-1*β* and TNF*α* in the peri-resection brain tissues. Immunoresponse plays a dual role in neuroinflammation process following a variety of neurological diseases [31–34]. The treatment strategies that regulate immune cells toward to anti-inflammatory phenotypes would benefit the ultimate outcomes [35–38]. The activation of microglial anti-inflammatory signaling of TGF-*β*1 by PS liposome prior to SBI insult provided neurological benefits in our experiment.

Notably, PS liposome improved the performance of modified Garcia test that evaluated composite neurological function after SBI. However, the neurological improvement was not evident in beam balance test. Previous study suggested that rats may compensate for deficits using the uninjured body side during neurological test [39]. The beam balance test examines the overall performance of a task, which may reduce the sensitivity to reveal unilateral brain damage in SBI, consistently to our previous findings [5].

We further investigated that the CD36 involvement in PS liposome provided anti-inflammation effects. The disruption of binding between PS liposome and CD36 offsets the anti-inflammation and neuroprotective effects of PS liposome against SBI. Knockdown of CD36 by siRNA potently attenuated the PS liposome-induced TGF-*β*1 protein production. Annexin V has been shown to have a high affinity to PS on apoptotic cells [40]. The application of rAnnexin V with PS liposome partially reversed the PS liposome-induced TGF-*β*1 protein production after SBI, most likely by attenuating the uptake of PS liposome by CD36. These findings echoed the previous in vitro study that apoptotic cells failed to induce TGF-*β*1 mRNA and protein production in macrophages after incubated with Annexin V [12].

Volatile anesthetics have been shown to disrupt the plasma lipid bilayer with resultant changes in plasma membrane phospholipid anatomy [41, 42], leading to translocation of PS from the inner leaflet to the outer leaflet of the plasma membrane. Such membrane externalization mimics the apoptotic signaling, promoting the release of the potent anti-inflammatory/antinecrotic molecule TGF-*β*1 via ligation of PS receptors in adjacent macrophages [12, 43, 44]. In naïve rats treated by isoflurane 1 hour/day for consecutive 3 days, there were significantly increased protein levels of CD36, p-Lyn, and TGF-*β*1 in brain tissues starting at 6 hours and up to 24 hours after the end of the pretreatment regimen. When the rats subjected to SBI subsequently, there were significantly less post-SBI neurological deficits and brain edema. In agreement with our results, Lee et al. previously reported that TGF-*β*1 released by volatile anesthetics mediated protection against renal proximal tubule cell necrosis [14]. The Lyn phosphorylation has been implicated as downstream signaling of the PS-CD36 initiated anti-inflammatory signaling involving in TGF-*β*1 induction [12]. Although exposure to volatile anesthetics for longer periods of time could induce programmed cell death [14], PS membrane externalization alone induced by short-term volatile anesthetic exposure was insufficient to fully execute the cellular apoptotic program and cell death [14]. Collectively, our results suggested that short-term isoflurane pretreatment regimen could result in the similar protective effects to PS liposome potentially activating the phagocytic signaling involving CD36/TGF-*β*1. Given the common clinical application of isoflurane, it bears a potential to be readily translated for patients undergoing neurosurgical procedure.

There are several limitations in the present study. The activation of PS recognition receptors other than CD36 may also participate in the PS liposome/isoflurane pretreatment-induced antineuroinflammatory effects. These different downstream signaling pathways need to be further investigated. The multiple mechanisms have been shown to underlie the neuroprotection provided by volatile anesthetic pretreatment, which were not fully elucidated in the study. In addition, we did not explore the gender and age difference in the antineuroinflammation effects provided by PS/CD36/TGF-*β*1 activation.

## 5. Conclusions

Our findings suggest that the activation of phosphatidylserine/CD36/TGF-*β*1 signaling pathway prior to SBI could attenuate neuroinflammation and improve neurological outcomes in rats. PS liposome or isoflurane pretreatment may serve as an effective preventive strategy to minimize the brain injury caused by neurosurgical procedures in patients.

## Data Availability

The data used to support the findings of this study are available from the corresponding author upon request.
